# Psychiatry and the Cinema for the Wellbeing and Development of Psychiatry Trainees: A Survey and Thematic Analysis

**DOI:** 10.1192/bjo.2024.328

**Published:** 2024-08-01

**Authors:** Tomos Jones, Kate Preston, Jonathan Stone

**Affiliations:** Avon and Wiltshire Partnership, Bristol, United Kingdom

## Abstract

**Aims:**

The importance of the humanities has been highlighted in developing a holistic person-centred model of psychiatry. The use of film to explore topics related to psychiatry, known as ‘cinemeducation’, has been shown to encourage reflection. Wellbeing has been identified as a key area in the quality of psychiatry training, however there is currently no evidence exploring the wellbeing and educational benefits of ‘cinemeducation’ within psychaitry training programmes

Our primary aim was to measure the impact of ‘cinemeducation’ events on attendees’ wellbeing and professional development, with a secondary aim to explore attendees experience of ‘cinemeduation’.

The hypothesis is that attendees will experience a wellbeing and educational benefit from the initiative.

**Methods:**

6 events were assessed between January and August 2023. Each event involved the showing of a feature length film, followed by a 30-minute discussion. 4 out of 6 events were facilitated by guest speakers, usually a consultant psychiatrist. Following events, questionnaires were distributed which included a series of statements with Likert scales and open ended questions. Mean Likert scale scores were calculated with qualitative data interpreted by the authors using thematic analysis.

**Results:**

A total of 108 trainees attended events, predominantly core trainees (64.52%). All events scored consistently high for self-reported wellbeing, however facilitated events demonstrated higher scores for self-reported reflective and educational benefits. The themes derived from qualitative data were of ‘cinemeducation’ being a *novel educational opportunity* where attendees were able to use film to work through challenges associated with psychiatry, as well as being an opportunity for *connecting with other trainees,* where attendees could share experiences and foster a sense of community.

**Conclusion:**

Core psychiatry trainees in particular, appear to value ‘cinemeducation’ as a tool to connect with their peers and develop their understanding of psychiatry in a relaxed, but stimulating environment, which is best achieved under the guidance of a senior colleague. The study suggests that the introduction of ‘cinemeducation’ across psychiatry training programmes would benefit trainees’ wellbeing and development. Further research is required to assess the impact of such initiatives across a broader cohort of trainees, using more robust methods of data collection, as well as formal measures of skills such as reflective functioning.

